# Distilling Artificial Recombinants from Large Sets of Complete mtDNA Genomes

**DOI:** 10.1371/journal.pone.0003016

**Published:** 2008-08-20

**Authors:** Qing-Peng Kong, Antonio Salas, Chang Sun, Noriyuki Fuku, Masashi Tanaka, Li Zhong, Cheng-Ye Wang, Yong-Gang Yao, Hans-Jürgen Bandelt

**Affiliations:** 1 State Key Laboratory of Genetic Resources and Evolution, Kunming Institute of Zoology, Chinese Academy of Sciences, Kunming 650223, China; 2 Laboratory for Conservation and Utilization of Bio-resource, Yunnan University, Kunming 650091, China; 3 Unidade de Xenética, Instituto de Medicina Legal, Facultad de Medicina, Universidad de Santiago de Compostela, Galicia, Spain; 4 Department of Genomics for Longevity and Health, Tokyo Metropolitan Institute of Gerontology, Tokyo, Japan; 5 Key Laboratory of Animal Models and Human Disease Mechanisms, Kunming Institute of Zoology, Chinese Academy of Sciences, Kunming, China; 6 Department of Mathematics, University of Hamburg, Hamburg, Germany; University of Montreal, Canada

## Abstract

**Background:**

Large-scale genome sequencing poses enormous problems to the logistics of laboratory work and data handling. When numerous fragments of different genomes are PCR amplified and sequenced in a laboratory, there is a high immanent risk of sample confusion. For genetic markers, such as mitochondrial DNA (mtDNA), which are free of natural recombination, single instances of sample mix-up involving different branches of the mtDNA phylogeny would give rise to reticulate patterns and should therefore be detectable.

**Methodology/Principal Findings:**

We have developed a strategy for comparing new complete mtDNA genomes, one by one, to a current skeleton of the worldwide mtDNA phylogeny. The mutations distinguishing the reference sequence from a putative recombinant sequence can then be allocated to two or more different branches of this phylogenetic skeleton. Thus, one would search for two (or three) near-matches in the total mtDNA database that together best explain the variation seen in the recombinants. The evolutionary pathway from the mtDNA tree connecting this pair together with the recombinant then generate a grid-like median network, from which one can read off the exchanged segments.

**Conclusions:**

We have applied this procedure to a large collection of complete human mtDNA sequences, where several recombinants could be distilled by our method. All these recombinant sequences were subsequently corrected by de novo experiments – fully concordant with the predictions from our data-analytical approach.

## Introduction

With the progress of large-scale genome sequencing in recent years, researchers are now beginning to explore the possibilities of detecting errors and improving the overall quality of sequencing results. For instance, numerous discrepancies between reported mRNA sequences and the July 2003 human genome sequence (for the 22 autosomes and two sex chromosomes) have been discovered [1], although an estimated upper bound of approximately four discrepancies for every 10,000 bases might not seem dramatic at first sight. Improvement on a program (base caller) that turns the fluorescent signal intensities detected by an automated sequencer into a DNA sequence could, for example, lower the error rate considerably [2]. The human genome project originally sought to attain an overall error rate of less than one error per 10,000 base pairs. If this error rate applied to the sequencing of the entire human mitochondrial genome comprising about 16,570 base pairs, then the majority of complete mtDNA sequences in a database would carry one or more incorrect bases – which was typically attained by the earliest sequencing attempts of the past, but most recent clinical mtDNA studies do not fare better and sometimes much worse [3]–[5]. Stipulating that two complete human mtDNA sequences sampled in some geographic region could typically differ in approximately 30 bases, then about 10% of the mismatches would be due to artefacts, under an error rate of 1∶10,000. Such an amount of errors, however, would be far too high for most medical and forensic studies of human mtDNA. For instance, in investigating whether specific mtDNA variation is associated with certain complex diseases [6], systematic sequencing errors can lead to spurious positive associations with the trait or erroneous speculations about the role of the mtDNA variants in the expression of the disease. In the forensic context, even a single error could be crucial in a case where the whole potential of the mtDNA genome would be explored [7]. There is some hope that complete mtDNA sequencing could reach a much higher quality level (by perhaps two orders of magnitude) when stringent lab routines are followed and overlapping sequencing of both strands is applied, such as may have been achieved with the data produced by [8].

After a phase of pioneering work of complete sequencing of the human mitochondrial genome in the past decade, new sequencing results, whether complete or partial, would no longer arrive in empty data space. Thus, every single datum can be compared to an analyzed database covering a good portion of the worldwide mtDNA variation. Since homoplasy in the coding region reaches only moderate levels for human mtDNA, estimation of an mtDNA phylogeny does in general not pose serious problems, quite in contrast to the situation with the limited information from the three hypervariable segments (HVS-I, HVS-II, and HVS-III) of the control region. In particular, the major branches (“limbs”) of the East Asian mtDNA phylogeny were already well documented a few years ago [9], [10], together carrying >95% of the major East Asian mtDNA haplogroups. Some deep branches that are quite rare and numerous younger branches (“twigs”) of the phylogeny are only beginning to emerge with more extensive sampling [11]–[14].

In principle, one can foresee three kinds of errors that may occur in virtually every mtDNA database: (1) sample mix-up or contamination (incurred during sample handling [4], [15]–[18]; (2) phantom mutations (arising through the sequencing process itself [19]–[23]; and (3) clerical errors and oversights of mutations (constituting a documentation problem [3], [24]. For a more detailed classification scheme of error patterns in mtDNA data, see [24]–[27]. The original coding-region data published by [28] constituted a prime example of mtDNA data affected by a considerable number of phantom mutations; see [29] for an announcement of correction.

Sample mix-up seems to be the most insidious source of error, which e.g. is mainly responsible for the dramatic situation with studies on seeming mtDNA instability in tumorigenesis [4] and in single-cell analysis [30]. To give another example, the SWGDAM forensic database [31], sponsored by the Federal Bureau of Investigation (FBI), comprising 4,839 combined HVS-I/HVS-II sequences, suffered from both clerical errors and artificial recombination [16], [17]. This database has been revised in a piecemeal fashion [32], [33], but documentation errors and artificial recombinants still persist [26]. Once sample mix-up affected a data set, mere re-reading of electropherograms would not be sufficient, but considerable efforts (including re-amplification and re-sequencing) are needed to cleanse the data from all potential artificial recombinants.

There are several strategies to detect anomalies in large data sets. Artificial recombinants of separate segments from different samples or other systematic errors could e.g. be discovered by the strongest reticulate signals in the data set through quartet-window analysis [34]. This method essentially is a character-based 4-taxon approach, where all quartets of taxa (haplotypes) are screened for incompatibilities between the parsimoniously informative sites relative to each quartet under investigation. A box network can visualize the thus distilled variation. The biggest boxes, reflecting the most extreme instances of mutual incompatibilities, can be expanded to more complex networks by comparing all haplotypes with respect to the same distilled set of sites that participated in the quartet box; see Fig. 11 of [35] and Fig. 6 of [34] for pertinent examples. The location of the haplotypes within the network may then suggest that an aberrant haplotype was generated through systematic errors such as sample mix-up rather than through natural homoplasy.

In a situation where sufficient data for comparison are available, a simple classificatory approach can help to detect a mosaic compound sequence stemming from different samples. In fact, idiosyncrasies of new data will mainly be manifest on the terminal branches connecting the new sequences to the mtDNA tree (for details, see below). Elson and Lightowlers proposed “to identify genomes with suspect combinations of markers that potentially indicate mtDNA recombination” [36]. First, the presence of certain characteristic mutations in a sequence suggests its approximate location in an mtDNA tree. Second, those mutations which are then seemingly private but do not ‘fit’ as they also occur *en bloque* in other parts of the mtDNA tree would suggest an alternative affiliation. If these ‘homoplasious’ mutations clustering on a single branch of the mtDNA tree are located within one or two amplicons, then we get a clear indication of artificial recombination. This can, for instance, be observed in Fig. 1A of [37]: the extremely long terminal branch of sample ND168 (from the haplogroup slot “M7a2”) exclusively carries recurrent mutations, six of which (all from the region 12406–14002) are also found along the pathway between sample TC20 (slot “F1a1”) and the tree root in Fig. 2 of [37].

**Figure 1:**
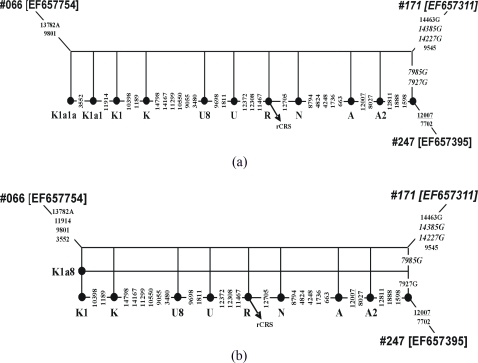
Two alternative networks displaying a reticulate pattern in the data of ref. 28 involving sequences #066 (GenBank Acc. No. EFF657754), #171 (EF657311), #247 (EF657395): network (a) displays the most plausible evolutionary pathways, whereas (b) would formally be obtained via the method described in this article applied to an up-to-date West Eurasian mtDNA classification tree. Note that only coding region data are in general available for this dataset, and therefore variation in the control region (such as the transition C295T diagnostic for K1a8) is not displayed in this network. The problematic haplotype (#171) and the two phantom mutations (C7927G and C7985G) are highlighted in italics. Solid nodes indicate the ancestral types of haplogroups along reconstructed evolutionary pathways; rCRS refers to the revised Cambridge reference sequence [39]. Mutations are prefixed and suffixed as usual [40].

**Figure 2:**
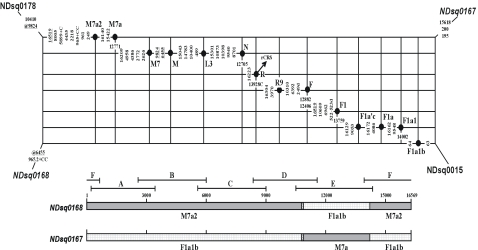
Network documenting sample crossover between NDsq0167 and NDsq0168. The diagram below the network indicates the readable regions of fragments A–F [37]; the bar is colored and labeled by those haplogroups with which the variation observed is consistent.

There is, however, a limitation to this approach when applied to a tree that is built upon existing data as well as a particular data set that comes under scrutiny. The inclusion of recombined sequences would slightly transform a tree, especially in the vicinity of multifurcation nodes. Any missed basal mutation could lead to a deeper branching and thereby distort the real temporal order of mutations without necessarily creating artificial homoplasy. Furthermore, if an “exchanged” mutation from the recombined segment stemming from another branch of the mtDNA phylogeny showed up on a terminal branch, the alarm bells would not ring because single recurrent mutations are frequently observed along terminal branches of any human mtDNA tree. Another difficulty is that exchanged fragments may stem from different continental samples which are used in different studies. A most recent discovery, concerning our own data, could identify a laboratory exchange of one segment (carrying two mutations, A4732G and G5147A) between the Ojibwa mtDNA sequence described in Table 3 of [38] and the Yakutian mtDNA sequence (GenBank Acc. No. DQ272125) [11]. Anyway, most laboratories have large sample collections from which only a subset is drawn for complete sequencing, so that an exchanged fragment may stem from a stored sample that was not targeted in the study.

To address such problems, we propose a formal strategy that can be carried out manually when reading freshly obtained sequences against a worldwide or continental mtDNA classification tree. For the sake of illustration, we apply this method to the mtDNA genome data reported by [37] which comprise 672 complete mtDNA genomes that were generated in a two-step PCR approach (the first PCR generated six long fragments, from which in a second PCR ten short overlapping segments were amplified each). The recombination instances we detected are reported here in the form of grid-like median networks [35], which are each generated by the recombinant sequence and the evolutionary pathway between the closest relatives of the two constituents of the sample mix-up approximated from the total database. Subsequent independent experiments finally confirmed that all the distilled recombinants are really artificial, further substantiating the validity of our approach.

## Methods

### Prerequisites

#### Classification tree and nomenclature

Any approach for allocating novel sequences to the hierarchical scheme of mtDNA haplogroups would start from a robust classification tree which focuses on the most basal branches and is estimated from previous data for the same geographic area or ethnic population under consideration. The nodes of such a classification tree *T* comprise reconstructed ancestral haplotypes inferred from a body of published complete mtDNA sequences. These haplotypes then constitute (typically unsampled) branching nodes of the total mtDNA tree, most of which are here referred to by letter-number strings (such as M7b1), designating the haplogroup with that particular root. Every mutation recorded in *T* is supported by at least two samples, so that private mutations of single sequences are disregarded. As usual, variation in human mtDNA is recorded relative to the revised Cambridge reference sequence (rCRS) [39]. We employed the updated nomenclature for East Asian mtDNAs provided by [11].

Since we wished to demonstrate how preliminary phylogenetic knowledge can assist in detecting recombinants in a large mtDNA data set, we took the classification tree derived from Fig. 1 of [10] prior to the publication of Tanaka et al.'s data, with three further corrections: first, the branch “C6” in that figure should be deleted as it is part of the Native American branch C1 and thus constitutes a misnomer; second, the 8027 transition needs to be added to the Native American branch A2 [38]; third, the 9180 transition is in fact shared by both branches (D5a and D5b) of D5 (as this mutation was missed in the originally recorded YN289). We are thus well aware of the omnipresent risk of inadvertent sample and data handling. Therefore the employed classification trees must be under permanent control since novel data sets could, in theory, challenge the reconstruction of the mutational history of previously sequenced samples.

#### Scoring

For every sequence from the classification tree *T* and any novel sequence under examination the shared mutations are evaluated. Counting mutations would be somewhat too simplistic because some mutations at certain positions may be very unstable (because of a site-specific mutation rate that is well above average) and therefore provide little evidence in favor of an artificial cause rather than natural evolution. We therefore adopt a coarse weighting scheme for mutations using scores 0, ½, 1, 2, and 3, according to Table 4 of [40], which is reproduced here as Table 1. The highest score, 3, is assigned to transversions in the coding region (577–16023) except for the relatively frequent event G13928C and to indels (insertions and deletions) except for C indels scored at position 965 and for indels hitting spacer regions between mtDNA genes. The lowest score, 0, is assigned to length polymorphisms of the two well-known C runs and the CA repeats located in the three hypervariable segments of the control region. The vast majority of substitutions in the control region have score 1 and transitions in the coding region have score 2, whereas a minority of ‘speedy’ transitions each receive half that weight. Some rare contiguous indels that are more complex may require *ad hoc* scoring as one or two character changes.

**Table 1:** Preliminary weighting scheme for mtDNA mutations [40]

Weight	Region	Type of Mutation	Mutation/Site/Fragment
**0**	HVS-I	C run length polymorphism	16182C, 16183C, C indels scored at 16193
	HVS-II	C run length polymorphism	C indels scored at 309 and 315
	HVS-III	Dinucleotide repeat	AC indels in 515–524 (alias CA indels in 514–523)
		C run length polymorphism	C indels scored at 573
**½**	HVS-I & 16519	Transition	16051 16078 16086 16092 16093 16111 16114 16124 16126 16129 16140 16145 16147 16148 16150 16163 16172 16173 16176 16186 16187 16189 16192 16193 16209 16212 16213 16214 16216 16217 16223 16227 16231 16232 16234 16235 16239 16240 16241 16242 16245 16249 16255 16256 16257 16258 16260 16261 16263 16264 16265 16266 16270 16274 16278 16284 16287 16288 16290 16291 16292 16293 16294 16295 16296 16298 16300 16301 16304 16309 16311 16316 16319 16320 16325 16327 16335 16352 16354 16355 16356 16357 16360 16362 16390 16519
		Transversion	16111A 16188A 16265C
		Indel	16166del
	HVS-II	Transition	93 146 150 151 152 182 183 185 189 194 195 198 199 200 204 207 228
	HVS-III	Transition	499
	Coding	Transition, indel	709, C indels scored at 965
**1**	Control	Any	All remaining mutations (not listed above)
	Coding	Transition	1438 1598 1719 1888 3010 3394 5147 5231 5460 5821 6182 6221 7055 8251 8790 9545 9554 9950 10398 11914 12007 12172 12501 13105 13359 13368 13708 13966 14110 15110 15217 15514 15924 15930
		Transversion	13928C
**2**	Coding	Transition	All remaining transitions (not listed above)
		Spacer indel	Indels within 3305–3306 4401 5577–5586 5656 5892–5903 7517 8270–8294 8365 14743–14746 15954
**3**	Coding	Transversion/indel	All remaining transversions/indels (not listed above)

#### Determination of Constituents in a Sample Mix-Up

The rationale for the multiple allocation procedure is that certain blocks of mutations express potential affinities of a mosaic sequence to different parts of the known mtDNA phylogeny. Since the quality of complete sequencing results is not known in advance and thus can vary across studies, we cannot stipulate that sequences under examination have always been well read and documented, so that multiple omissions of mutations, for whatever reason, should be anticipated (see e.g. [3]). Such systematic oversights would act as a virtual crossover with the rCRS in the reading and documentation process. Further, we cannot know in advance how many constituents a mosaic sequence possesses. Therefore, in a first round, we recognize the broad haplogroup status of a sequence under scrutiny and then determine its primary constituent that could cover/explain most (weighted) mutations. In a second round, we then exclusively consider the mutations not yet covered, some of which (in conjunction) could point to a different haplogroup affiliation and thus serve as positive evidence for the existence of a secondary constituent. In a third round, we seek for evidence that blocks of specific mutations of the primary constituent are missed, given its affiliation in the tree.

The allocation process then has two (or more) iterations. In the first iteration, one seeks a best allocation of the novel sequence to a node of the classification tree *T* that explains most of the (weighted) mutations relative to rCRS and subsequently searches for a closest representative beyond this ancestral sequence in the total database (including the new data under examination). This, in the case of a recombinant event, will determine the primary constituent of the potential sample mix-up as represented by a closely related sequence that is available in the total database. Then, in the second iteration, one searches for another node in the tree *T* which covers most of the remaining “private” mutations in the novel sequence not yet covered by the first putative constituent. This will point to the second constituent of the potential mix-up. If necessary, one could continue the search for a third constituent covering part of the yet unexplained variation relative to the reference sequence, but the recombination events we have encountered so far seem to involve only two clearly discernible constituents.

#### Primary Classification

For every novel sequence *s* under examination we search the relevant classification tree *T* for (ancestral) sequences represented by nodes that cover the maximum score of mutations of *s* relative to rCRS. Among those ancestral nodes attaining the maximum score we select one, *t*, that determines a path to the rCRS minimal with respect to inclusion. If more than one minimal choice was possible, the alternative(s) would be stored and explored as well. Then we search the total complete mtDNA genome database for any further sequence(s) *u*, for which the evolutionary pathway connecting *u* to the rCRS would pass through *t* so that additional mutations (if present) seen in *s* could be covered by *u*.

#### Secondary Classification

The mutations of *s* which are not yet covered by the first companion *u* are screened in the classification tree as in the preceding step. Hence we determine another ancestral node *t*' from the classification tree that captures the maximum score of those remaining mutations of *s* and determines a minimal path to the rCRS. Then, as before, we screen the total database for any further sequence(s) *u*' beyond *t*', for which the evolutionary pathway connecting *u*' to the rCRS would pass through *t*' so that as many of the “private” mutations finally left in *s* as possible are covered by *u*'. The remaining unmatched mutations would then indicate private variants (generally assigned to the major constituent), unless a tertiary classification would capture a good share of the variants not yet covered.

#### Thresholds

Since homoplasy may confound the classification of novel sequences, we apply a moderate threshold for allocation to nodes of the classification tree in order to avoid hypersensitivity of the method. The threshold has to be adjusted to the task in question and may be chosen dependent on the structure of the tree in the vicinity of each node. Here we propose a score 2 threshold for the first round of the secondary classification. This implies, in particular, that the presence of a single homoplasious transition in the control region would not be enough to pursue the search. That is, we scan the database beyond a particular node from the classification tree only when score 2 is reached for the mutations covered that far. After scanning, we then require that the secondary constituent of a potential recombination instance cover mutations of total score 3 or higher. Thus, a single coding-region transition would not suffice at this stage. For internal lab routines executed on batches of freshly obtained sequences, one may of course handle a lower threshold in order to increase the sensitivity of this kind of *a posterior* control (at the expense of ‘unsuccessful’ re-reading and re-sequencing efforts).

The strength of a recombination instance is finally expressed by the minimum of the total scores of ‘false’ nucleotide variants relative to the primary and secondary constituents. For a clear-cut recombination instance we would require a total score at least 6. This score can be read off from the graphical display (see below) by adding up the scores of mutations (that do not cancel each other) along the vertical links – provided that the dominant constituent is scored by the mutations along the links drawn horizontally.

### Graphical Display

#### Pathway between constituents

We reconstruct the pathway *P* between the two approximate constituents *u* and *u*' according to the total tree, capturing all intermediate branching nodes from the tree. The role of the branching points is to interrupt the otherwise arbitrary order of mutational events separating the two constituents; mutations attached to a single link, however, remain unordered; for convenience, they are listed in the natural order. In a diagram we label each node by the code of the haplogroup for which the corresponding sequence is the most recent common ancestor. The point to which the rCRS has to be attached is highlighted.

#### Median network

For any set *S* of aligned binary sequences, the median network comprises as nodes all sequences that can be successively generated from *S* by taking majority consensus, so that the links constitute the minimum spanning network for the total node set [35], [41]. The links are labeled by the mutational events. In the case of the evolutionary path *P* between the approximate constituents *u* and *u*' of a putative recombinant *s*, the set *S* is formed by *s* and all nodes of *P*. Since the mutations labeling the links of *P* are reconstructed events, any recurrent mutations are formally treated as mutations hitting different sites [41]. The median network generated by this particular set *S* has a simple two-dimensional structure: namely, it is a “half-grid” for which the mutations on the horizontal line segments correspond to the mutations of the recombinant *s* covered by the primary constituent *u*, whereas the mutations along the vertical links are those covered by the secondary constituent *u*'. Private mutations for *u*, *u*', or *s* are allocated to the corresponding terminal links.

#### Reconstruction of the Recombination Event

Before we may infer that artificial recombination acted upon the sample under examination, it is desirable to find a simple explanation for the apparently false mutations. First, the mutations must be allocated to the (overlapping) fragments/segments of the particular sequencing procedure. For a two-step PCR approach (as used by [37]), we would highlight only the long fragments of the first PCR analysis provided that the exchange of whole fragments testifies to some sample mix-up and would thus explain the recombinant pattern. Segments of the second PCR analysis (especially covering overlap regions of two fragments) are highlighted whenever they show a pattern deviating from the remainder of the involved fragments/segments. Such exceptions would then require some explanation. Especially for the cases (obtained in the second round) where the evidence for potential sample mix-up is dominated by the missed mutations, we need a convincing exchange pattern that characterizes this case as a recombination instance and distinguishes it from mere oversight of mutations due to poor base calling or inadvertent documentation. Finally, following a parsimonious principle, we would aim at merging two recombination instances into one crossover instance whenever possible (by substituting potential constituents yielding nearly the same score – if necessary).

## Results

### Phantom Mutations versus Recombination

To give an example of a median network generated from a single sequence together with an evolutionary pathway between two other sequences, take the mtDNA sequence #171 and the pathway between sequences #066 and #247 from the uncorrected data set of [28]. These sequences can still (as of December 2007) be found in the mtDB database (http://www.genpat.uu.se/mtDB/) by searching for the mutations C13782A (#66), T14463G (#171), and G7702A (#241), and then by downloading the corresponding sequences directly via GenBank (#66 = EF657754, #171 = EF657311, and #247 = EF657395). Sequence #066 is a member of the West Eurasian haplogroup K1a1a [42], whereas sequence #247 belongs to a subhaplogroup of the Native American haplogroup A2 characterized by three mutations (at positions 1598, 1888, and 12811). Figure 1a displays, in particular, the evolutionary pathway between #66 and #247, which descends through a nested array of ancestral haplotypes (marked in the figure by the corresponding haplogroup names) from the K1a1a type down to the root of haplogroup N and then ascends to the particular A2 haplotype. Sequence #171 is closely related to sequence #247 but shares the transversion pair C7927G and C7985G with the unrelated sequence #066. This leads to ladder-like reticulation that runs through the entire non-private part of the evolutionary path.

With respect to our scoring scheme, the vertical part of the network of Fig. 1a weighs 6 units, thus indicating a conspicuous reticulate signal. Nonetheless, this instance would not pass through the first stage of our primary classification procedure because no West Eurasian mtDNA classification tree whatsoever would offer a link labeled by C7927G and C7985G. This is well and good, because this case would not constitute a recombination instance but rather document the action of a phantom mutation process, where artificial mutations were repeatedly inflicted on phylogenetically unrelated sequences. In the revised data [29], these and other mutations disappeared; see also Table 1 of [40] for the fate of six C to G phantom transversions that were frequent in the original data set. However, the old data still survive not only in the author's web repository (http://mtsnp.tmig.or.jp/mtsnp/search_mtDNA_sequence_e.html) and in recent publications (e.g. [42]), but also persist in the mtDB database and the very recent GenBank version (EF657231 to EF657790; submitted in June 2007).

Nonetheless, if we would use an elaborate West Eurasian classification tree which improves upon the one given by [43] by incorporating, for instance, a (corrected) version of the haplogroup K tree from [42], then, most astonishingly, we get a candidate for the secondary classification from haplogroup K1a8: this haplogroup has the two characteristic mutations C295A and C7927G! This secondary constituent then yields score 6.0 and would thus support a seeming recombination instance; see Fig. 1b. In reality, of course, we are seeing here the effect of an almost unbelievable coincidence: on the one hand, C7927G is a confirmed phantom mutation that has affected several sequences from [28], and on the other hand, it acts as a real mutation defining a minor subhaplogroup of haplogroup K1a.

### A Paradigmatic Case of Sample Crossover

The 672 complete mtDNA sequences of [37], as downloaded from the website http://www.giib.or.jp/mtsnp/search_mtDNA_sequence_e.html on November 25, 2004 and now also available in GenBank, were subjected to a thorough *a posteriori* quality checking. We refer to all samples by their original names listed on the website. We took care that obvious editing errors such as seeming insertions of the kind 16569+G and 16569+GATCACAG do not enter our analyses. Based on our classificatory approach (see Methods), a total of nine recombinants with score at least 6.0 could be identified (Table 2), two of which can be regarded as partners in a sample mix-up.

**Table 2:** Recombinants detected in published complete mtDNA genome data

Recombinant (GenBank Acc. No.)	Haplogroup/Closest Relative		Fragment or Segment Involved		Deviant Segment	False Variants	
	Primary	Secondary	1^st^ PCR	2^nd^ or Direct PCR		Count	Score
NDsq0167 (AP008798)	F1a/NDsq0015	M7a/NDsq0178	E (11204-14141)			7	13.0
NDsq0168 (AP008799)	M7a/NDsq0178	F1a/NDsq0015	E (11204-14141)			8	15.0
NDsq0181 (AP008803)	M8a2a/Kong#WH6958	D4b2b1/HNsq0221	A (121-3036)			3	6.0
NDsq0116 (AP008776)	D4e2/JDsq0080	D5b/TCsq0030	D (8366-11330)			5	10.0
NDsq0117 (AP008777)	D5b/TCsq0030	D4l/JDsq0100	D (8366-11330)			5	10.0
ONsq0025 (AP008552)	B5a2/NDsq0126	D4g1a/ONsq0035	E (11204-14141)			4	8.0
GCsq0033 (AP008259)	D5a2a/PDsq0097	A/TCsq0048		57-60 (15717-355)		14	8.5
TCsq0010 (AP008269)	M7a1b/TCsq0007	B4c1c/ONsq0039	A (121-3036), B (2885-5782), C (5623-8482), E (11204-14141)		30	16*	28.0*
TCsq0019 (AP008278)	D4j/NDsq0124	M7b2/NDsq0165	B (2885-5782)		11,18	6	11.0
Young#BJ109 (N.A.)	D4b2b/Macaulay#2	M10a1/Kong#YN163	No. 6, 18, 19, 22 according to [45]		21	10	18.0

Note: The first nine cases are drawn from [37] and the last one from [46]. The samples employed for comparison are from [37], except two from [10], [13]. The readable regions of the segments mentioned for the first nine cases are as follows [44]: 11 (2885-3284), 18 (4848-5247), 21 (5623-6022), 30 (8083-8482), 57 (15717-16116), 58 (15969-16368), 59 (16221-51), and 60 (16525-355). False mutations are rated with respect to the primary classification except when the secondary classification gave a smaller score (indicated by an asterisk). N.A. = not available.

As shown in Fig. 2, the primary constituent of NDsq0168 comprises most of the mutations characteristic of haplogroup M7a2 (except C12705T and G12771A), whereas the secondary constituent of the sample encompasses five seemingly “private” variants (viz. G12406A, C12882T, G13759A, G13928C, and A14002G) which clearly point to haplogroup F1a1b. The relatively high score (viz. 9.0) of the secondary constituent coupled with the fact that the involved five F1a1b specific mutations are all located within one fragment (viz. fragment E, with readable region 11204–14141 [44]) generated at the first PCR stage, constitute a salient signature that the reported fragment E in NDsq0168 was accidentally taken from some haplogroup F1a1b sample. This exchange of fragments also well explains the absence of the expected mutations C12705T and G12771A in the haplogroup M7a2 sample. Coincidentally, an exactly opposite pattern is observed in sample NDsq0167 – the predecessor of NDsq0168 in the ND series. Namely, the primary constituent of NDsq0167 encompasses most of the mutations characteristic of haplogroup F1a1b (with the exception of G12406A, C12882T, G13759A, G13928C, and A14002G), whereas the secondary constituent of the sample consists of two “homoplasious” mutations, C12705T and G12771A (score 4.0), which occur on the pathway to haplogroup M7a.

The total scores of all false mutations are then 13.0 and 15.0 for NDsq0167 and NDsq0168, respectively. These scores differ because the transition at 6455 is missing in NDsq0168 but is not present in NDsq0167 either. Nonetheless, the evidence is most compelling that fragment E was interchanged between samples NDsq0167 and NDsq0168, thus constituting a clean case of sample crossover.

### ‘Data Mining’ of Artificial Recombinants

**Figure 3:**
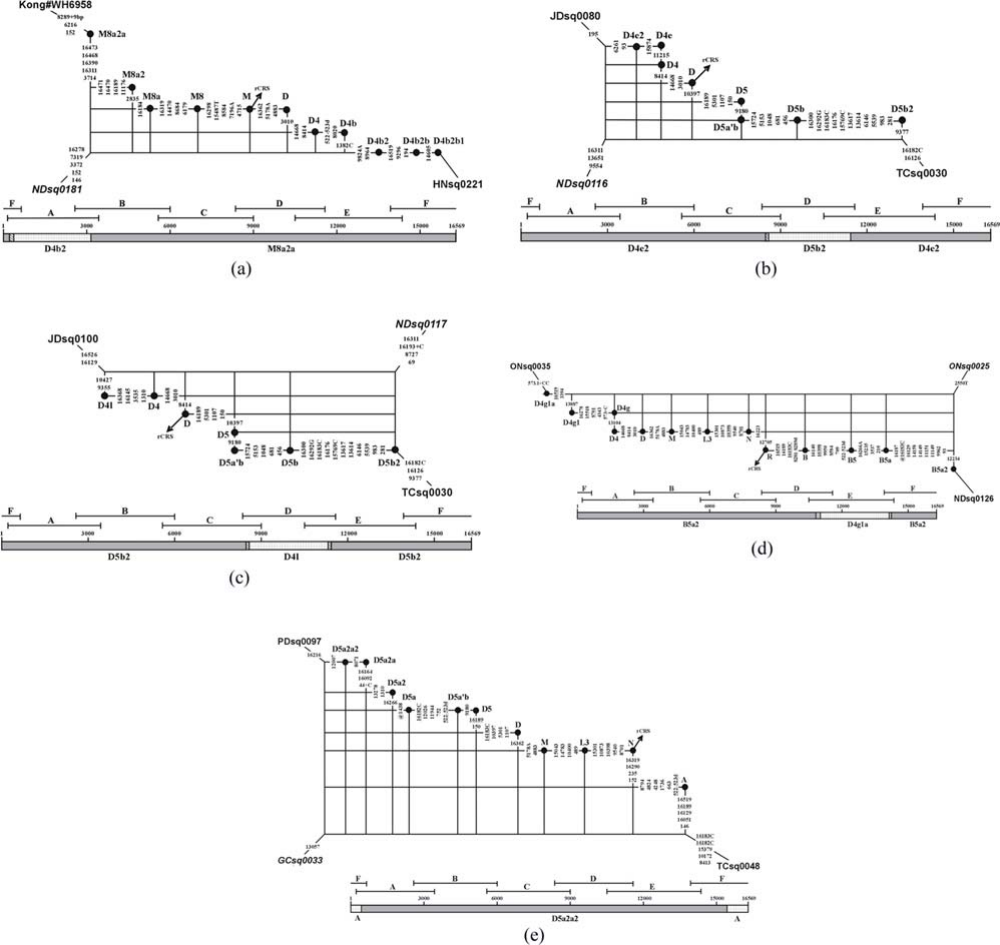
Networks for six recombinant sequences in which only one fragment or segment each was exchanged with some other (unknown) sample: (a) NDsq0181, (b) NDsq0116, (c) NDsq0117, (d) ONsq0025, and (e) GCsq0033.

**Figure 4:**
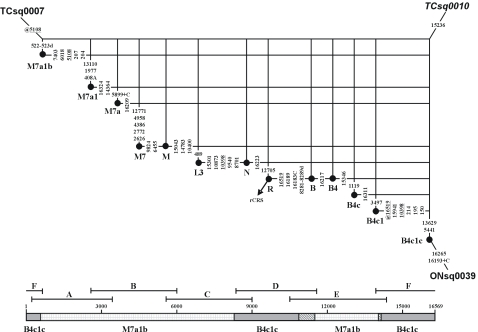
Network for the recombinant sequence TCsq0010 in which multiple fragments were exchanged as indicated along the bar.

For most of the putative recombinants detected in the 672 complete mtDNA sequences reported by [37], the corresponding secondary constituents each involved the exchange of a single fragment generated at the first PCR stage (Table 2), such as fragment A in NDsq0181 (Fig. 3a), fragment D in NDsq0116 (Fig. 3b) or NDsq0117 (Fig. 3c), fragment E in ONsq0025 (Fig. 3d), or the part of fragment F covering HVS-I and HVS-II in GCsq0033 (Fig. 3e). The exception is TCsq0010 (Fig. 4), in which several fragments have been exchanged. As indicated by its primary constituent, TCsq0010 belongs to haplogroup B4c1c, although two B4c1c diagnostic mutations (viz. at sites 1119 and 3497) are missing. The secondary constituent, consisting of 11 mutations mainly located in fragments A, B, and E, clearly suggests M7a status. The extremely high score (viz. 28.0) of the involved mutations strongly supports the notion that the current fragments A, B, and E in TCsq0010 were interchanged from an M7a1 sample, which then resulted in the loss of mutations T1119C and C3497T characteristic of haplogroup B4c1.

**Figure 5:**
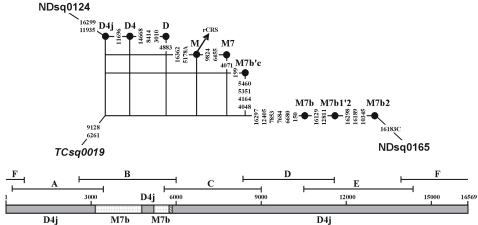
Network for the recombinant sequence TCsq0019 where one deviant segment from the exchanged fragment had likely been sequenced independently.

The situation in TCsq0019 seems to be more complicated. As shown in Fig. 5, the primary constituent of this sample supports haplogroup D4j status [37], but its secondary constituent harbors five additional mutations at sites 4048, 4071, 4164, 5351, and 5460 (with total score 9.0) which are all specific to haplogroup M7b. According to the preceding strategy, it seems obvious that fragment B (readable region: 2885–5782), which covers the aforementioned five sites, must have been exchanged from an M7b sample. This well explains the absence of mutation C4883T in the sample but not the presence of mutations G3010A and C5178A in the same fragment, which are diagnostic for haplogroup D4. Since C5178A is a very rare mutational event, it is rather implausible that the two occurrences of the variant nucleotide in the sample could simply be attributed to natural parallelism. According to [44], fragment B was re-amplified into ten shorter overlapping segments (viz. 11–20) at the second PCR stage, the five M7b mutations are then mainly located in segments 13, 14, 15, 17, 19, and 20, whereas the two D4 mutations are in segments 11 and 18. This exceptional pattern reflects the minimal correction procedure that was originally carried out: some segments (e.g. 11 and 18) or much shorter ones were regenerated after the complete sequencing was carried out because of (1) an obvious conflict between the absence of some well-known haplogroup-diagnostic mutation (such as C5178A) and a certain familiar HVS-I sequence motif, or (2) the conflicting information at the same position (e.g. 3010 covered by both fragments A and B) in the overlapping region of neighboring fragments. Direct re-amplification of a single short segment would then have constituted the most straightforward strategy for reconciling such conflicting results. This explanation could also apply to the presence of 8281–8289del in the haplogroup B4c1c sample TCsq0010, for which fragment C otherwise perfectly conforms to haplogroup M7a1b status.

### Recombination in a 24-Segment Amplification Set-up

**Figure 6:**
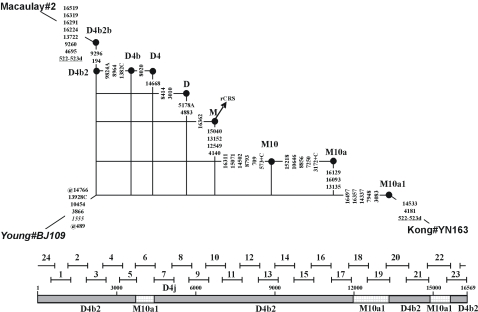
Network featuring the recombinant type BJ109 from [46]. The diagram below the network visualizes the sequencing ranges of the employed 24-segment amplification setup ([45]; Table 1); the bar is labeled by the haplogroups D4b2 and M10a1 with which the variation recorded for BJ109 is consistent. The two mtDNA samples for comparison are from [10], [13], respectively. The pathogenic mutation A1555G is highlighted in italics.

Any inadvertent exchange of a large fragment of more than 3.0 kb stands a very good chance to leave a trace manifest as a reticulate signal. However, with segments of 0.5 to 1.0 kb that are directly amplified and sequenced there is a considerably lower chance that recombination leaves a well detectable fingerprint. The popular set of 24 primer pairs proposed by [45] is of this kind. Nevertheless, under favorable circumstances, e.g. when several characteristic mutations cluster in one segment, clear evidence for recombination may show up in an *a posteriori* analysis. This is the case with sample BJ109 sequenced by [46]. This mtDNA sequence predominantly has haplogroup D4b2 status, but a secondary signal for haplogroup M10a1 is quite pronounced (Table 2 and Fig. 6). In this case, evidently more than one segment had been taken from a sample with haplogroup M10a1 status, which thus reinforces the inference of sample mix-up [5].

The Rieder et al. 's primer pairs were also employed by [47] at the time. Fig. 1 of [40] displays these data in a tree with mutational events reconstructed. Since recurrent mutations are highlighted there, one can also visually inspect the tree for repeated combinations of mutations. This leads one to suspect that one Biaka sequence from haplogroup L1c1a had its back mutations of transversion T4454A (designating L1c1a status) and transition A3843G (designating L1c1 status) inherited from the haplogroup L1b or haplogroup L1c2a sample of the same data set. In fact, sample confusion involving segment 6 (Table 1 of [45]) could explain the simultaneous loss of those two mutations, without creating any further false mutation. Using our scoring system this potential recombinant would yield score 5.0. Another potential recombinant with score 4.0 could be the Saami sequence from haplogroup V in that data set, for which segment 21 might have been exchanged with the Papua New Guinea (coast) sequence from haplogroup P1a: such an exchange could explain the back mutation at site 14766 and the parallel mutation at site 14097. However, one cannot firmly exclude the possibility in this case that this event could be due to natural homoplasy.

### Substantiation of the Artificial Recombination Events

To substantiate the reconstructed artificial recombination events and thus the validity of our data-analytical approach, independent experiments (including PCR re-amplification and DNA re-sequencing following the same protocol as in [37]) were eventually applied to all the suspicious fragments/segments of the 9 recombinants distilled from the 672 complete mtDNAs [37]. Table S1 (Supplementary Material) summarizes the novel sequencing results, where the corrected variation of the involved fragments/segments is contrasted with the previously reported variation. The re-sequencing analysis demonstrated that all the suspicious fragments/segments were the product of artificial recombination events – exactly as predicted by our data-analytical approach.

All of the candidate recombinant sequences from that data set with score 6.0 or higher had thus a plausible explanation by exchange of at least one fragment. Since some fragments would not strongly discriminate between different samples, one cannot expect that the total score of exchanged mutations always reaches 6.0. We thus have to count with further, nearly silent recombination events that have affected the data from [37]. We suspected that samples GCsq0016, TCsq0049, TCsq0047, and NDsq0178 were also affected by sample mix-up in view of some signals of recombination, which are though not sufficiently conclusive on their own. Unfortunately, DNA from the former two samples is no longer available, so that we must content ourselves with analyzing the latter two cases.

Sample TCsq0047 lacks two mutations with total score 4.0 (C12633T and C12882T) characteristic of haplogroup F1b, which are located in the same amplicon (viz. segment 46, with readable region 12623–13022 [44]). However, an oversight of these mutations due to reading difficulties could equally have generated such a pattern. Re-sequencing of fragment E for this sample eventually revealed that C12633T and C12882T were indeed missed and A12358G was erroneous, so that some sample mix-up is now plausible (say, involving PDsq0139) under the hypothesis that the well-known haplogroup F1 marker G12406A had been established beforehand.

Sample NDsq0178 constitutes an almost perfect haplogroup M7a2 sequence – if there were not the haplogroup M7 mutation T9824C absent and the haplogroup D4a1 marker T10410C present, which together yield total score 4.0. However, re-sequencing of fragment D (8366–11330) gave a surprising result: not only were the two mutations conjectured as false confirmed as real but in addition were the mutations C8414T (characteristic of haplogroup D4) and T8473C (characteristic of haplogroup D4a) detected as well. This brings the total score of false mutations up to 8.0. Since the latter two mutations are normally also covered by fragment C (5623–8482), they were apparently not reported originally. We are thus faced with the unanticipated situation that five fragments (A, B, C, E, and F) of NDsq0178 came from the wrong sample. This could be confirmed by subsequent re-sequencing of the entire mtDNA genome of NDsq0178: the sample was finally proven to belong to haplogroup D4a1 as originally indicated only by fragment D (Table S1).

The most likely source of the five exchanged fragments of NDsq0178 might come from NDsq0168 (sharing several highly specific mutations with NDsq0178), which was itself involved in sample mix-up. The only two differences in the haplogroup M7a2 portions of the originally published sequences NDsq0168 and NDsq0178 constitute the lengths of the elongated C run from position 956 beyond 965 and the transition at position 6455. The former difference is insignificant because both length variants (965+2C and 965+4C) can coexist because of pronounced heteroplasmy. The latter difference could be resolved: it now turned out that C6455T is well present in sample NDsq0168. Therefore the complex sample interchange can now be elucidated as follows: the true sample NDsq0178 was a haplogroup D4a1 sequence that was predominantly confused with the true haplogroup M7a2 sample NDsq0168. The latter sequence was originally corrupted by an unknown sample belonging to haplogroup F1a1b. Then the sequence NDsq0167 (mainly reflecting haplogroup F1a1b membership) received a fragment from the true sample NDsq0168.

## Discussion

The search for artificial recombination constitutes a necessary step before the utilization of mtDNA data, but its detection is not a trivial task. With the manual procedure proposed in the present study, some artificial recombination events could be distilled effectively by using a given classification tree, a scoring system for mutations, and the median network tool. To facilitate the analysis of large-scale genomic mtDNA data sets, we are developing a corresponding automatic tool (which will be elaborated on in a subsequent paper). However, it is worth noting that the ability to detect errors in complete genome data sets by using this phylogenetic methodology will be limited, to some extent, by the currently available data and the resolution of the mtDNA phylogeny. To give an example, suppose that we were given the most recent detailed East Asian classification tree from [11] and would now, with hindsight, compare the originally published haplogroup M10a1 sequence YN163 (Fig. 1 of [10]; GenBank Acc. No. AY255178.1) to this mtDNA tree. Then, very clearly, we would allocate this sequence to the correct haplogroup but observe that transitions G13135A and A13152G were missing. Now, when searching for a potential secondary constituent we would get absolutely no signal because the seeming private mutations (at positions 4181, 13269, and 14533) are not present in this East Asian classification tree. However, a thorough search in the same data set [10] reveals a sequence from haplogroup R11, namely QD8168 (GenBank Acc. No. AY255163), bearing the private variant at 13269. Employing this R11 sequence as a secondary constituent would then yield score 6.0 for this recombination instance. Thus the price for having a robust method for inference of recombination that does not heavily rely on single entries in the total database is that some recombination instances may slip through the sieve of secondary classification.

Another limiting factor in a direct multi-segment amplification set-up is the low number of mutations that a single segment may carry. For example, the sample crossover described in the *Introduction* between the haplogroup X2a sequence (sampled from the Ojibwa) and the haplogroup F1a1 sequence (from the Yakut) led to a displacement of two transitions (A4732G and G5147A) of total score 3.0 from the latter to the former sequence. It is thus profitable to investigate any potential recombination instance where the score reaches a lower threshold, say, 3.0 – half of the threshold we have applied to the data of [37].

Nowadays, complete mtDNA genome sequencing has become a popular approach in human evolution, medical and forensic genetics studies. Numerous problems detected in some of the reported genome data sets (cf. [4], [11], [43], [48], [49]; this study) indicate that the normal lab routines and standard data analyses are far from sufficient to guarantee fully reliable sequencing results. There are several reasons why complete sequencing of the mitochondrial genome could give erroneous results. First, insufficient reading and documentation of the sequencing results could induce numerous oversights, so that typically mutations are missed, as in the case of the data from [50], [51]; for more details see [43], [48], respectively. Second, the sequencing process itself could introduce phantom mutations in a considerable number of mtDNAs [23], [28], [29]. Third, inadvertent editing of the sequences would lead to some obvious errors, such as 16569+G and 16569+GATCACAG in [37]. Fourth, due to contamination or sample mix-up, the assignment of amplified fragment/segments to a compound complete sequence could produce artificial recombinants. Unfortunately, the latter source of error persists in recent complete sequencing studies of human mtDNA, especially in the medical field [3]–[5], which would then fatally devaluate a huge amount of invested work. Note that data obtained using the human MitoChip [52] also had the above-mentioned problems [15], although in theory the possibility of sample crossover – but not of contamination – should be lower, as only one sample is screened by one chip.

Therefore, very strict lab routines need to be implemented in order to make sample confusion highly unlikely. In particular, strategies of long-length overlapping sequencing of fragments have to be employed by means of direct PCR [53]. It goes without saying that any experimental study that claims to have detected mtDNA recombination has to report the set of employed primer pairs alongside with the results – but unfortunately, this minimal requirement is not always met by high-rank journals (as in the case of the extraordinary claims by [54]). The analysis of mtDNA data also has to incorporate the up-to-date knowledge of the worldwide mtDNA phylogeny so that every single new sequence can be compared to its closest mtDNA relatives. In this spirit, our strategy of *a posteriori* analysis of mtDNA data, which pinpoints the potential recombinants by visualizing a reticulate pattern, will be useful in detecting the artificial recombination events and thus helps minimizing errors that could otherwise lead to undesirable consequences.

For the time being, one has to anticipate an omnipresent thin layer of artificial variation incurred by sample mix-up. For the entire database encompassing more than 2,900 complete and 900 semi-complete (coding) human mtDNA sequences we would propose a conservative estimate of 1% of recombined sequences where at least one segment was exchanged. More pessimistically, one could hold the view that the recombination rate (per sequence) was as large as 3%, say. There is no reason to assume that other databases, e.g. for animal mtDNA, are less prone to sample mix-up. Since the total number of entire mtDNA genome sequences per species is much lower than for humans, inadvertent sample mix-up or contamination would be more difficult to spot in the laboratory by comparison with the body of existing data, and consequently, the artificial recombination rate may even be higher. It is then certainly discomforting when studies that promulgate widespread recombination of animal mtDNA do not take the most natural source of seeming recombination, laboratory artifacts, into consideration [55].

## Supporting Information

Table S1 Correction of erroneous sequences from ref. [37] by re-sequencing or re-reading.
(0.05 MB DOC)
