# Supplementary material for: Distilling Artificial Recombinants from Large Sets of Complete mtDNA Genomes
Source: PLoS One. 2008 Aug 20;3(8):e3016. doi: 10.1371/journal.pone.0003016 (PMC2515346; doi:10.1371/journal.pone.0003016)
Supplement: Table S1 — Correction of erroneous sequences from ref. [37] by re-sequencing or re-reading. (0.05 MB DOC) [file pone.0003016.s001.doc]

**Supplementary Material**

**Table S1. Correction of erroneous sequences from [37] by re-sequencing or re-reading**

| Erroneous Sequence  (GenBank Acc. No.) | Re-sequenced Fragment(s) / Segment(s) | Previous Variation in the Fragment(s) / Segment(s) | Corrected Variation |
| --- | --- | --- | --- |
| NDsq0167 (AP008798) | E (11204-14141) | 11719 12705 12771 | 11719 12406 12882 13759 13928C 14002 |
| NDsq0168 (AP008799) | E (11204-14141) | 11719 12406 12882 13759 13928C 14002 | 11719 12705 12771 |
| NDsq0181 (AP008803) | A (121-3036) | 146 152 263 309+C 315+C 489 522-523d 750 1382C 1438 2706 3010 | 146 152 263 309+C 315+C 489 522-523d 750 1438 2706 2835 |
| NDsq0116 (AP008776) | D (8366-11330) | 8701 8860 9180 9377 9540 9554 10397 10398 10400 10873 | 8414 8701 8860 9540 10398 10400 10873 11215 |
| NDsq0117 (AP008777) | D (8366-11330) | 8414 8701 8727 8860 9355 9540 10398 10400 10427 10873 | 8701 8860 9180 9377 9540 9554 10397 10398 10400 10873 |
| ONsq0025 (AP008552) | E (11204-14141) | 11719 12705 13104 13887 | 11719 12234 |
| GCsq0033 (AP008259) | 57-60 (15717-355) | 16051 16129 16182C 16183C 16189 16223 16290 16319 16519 73 146 152 235 263 309+CC 315+C | 16092 16164 16182C 16183C 16189 16223 16266 16362 16519 44+C 73 150 263 309+C 315+C |
| TCsq0010 (AP008269) | A (121-3036)  B (2885-5782)  C (5623-8482)  E (11204-14141) | 408A 489 522-523d 750 1438 1977 2626 2706 2772  4386 4769 4958  5899+C 7028 8281-8289d  11719 12705 12771 13110 | 750 1007 1119 1438 2706  3497 4769 5441  7028 7752 7870 8281-8289d  11719 13629 |
| TCsq0019 (AP008278) | B (2885-5782) | 3010 4048 4071 4164 4769 5178A 5351 5460 | 3010 4769 4883 5178A |
| TCsq0047 (AP008306) | E (11204-14141) | 11719 12358 12406 13928C | 11719 12406 12633 12882 13928C |
| NDsq0178 (AP008801) | A (121-3036)  B (2885-5782)  C (5623-8482)  D (8366-11330)  E (11204-14141)  F (14007-355) | 73 249 263 309+C 315+C 489 750 961 965+CC 1438 2218 2626 2706 2772  4386 4435 4769 4958  5899+C 6455 7028 8005  8701 8860 9540 10398 10400 10410 10873  11719 12705 12771  14766 14783 15043 15301 15326 15422 16140 16209 16223 16519 | 73 146 152 263 309+C 315+C 489 750 1438 2706  3010 3206 4313 4769 4883 4959 5178A  7028  8414 8473 8701 8860 9540 10398 10400 10410 10873  11719 12705  14467 14668 14766 14783 14978 14979 15043 15301 15326 16223 16362 16519 |
